# Living with opioids: A qualitative study with patients with chronic low back pain

**DOI:** 10.1111/hex.13089

**Published:** 2020-06-18

**Authors:** Helena De Sola, Amaia Maquibar, Inmaculada Failde, Alejandro Salazar, Isabel Goicolea

**Affiliations:** ^1^ The Observatory of Pain University of Cádiz Cádiz Spain; ^2^ Biomedical Research and Innovation Institute of Cádiz (INiBICA) Cádiz Spain; ^3^ Preventive Medicine and Public Health Area Cádiz Spain; ^4^ Department of Nursing I, Faculty of Medicine and Nursing University of the Basque Country UPV/EHU Leioa Bizkaia Spain; ^5^ Department of Statistics and Operational Research University of Cádiz Cádiz Spain; ^6^ Department of Epidemiology and Global Health Umeå University Umeå Sweden

**Keywords:** biomedicalization, chronic pain, experience, low back pain, opioid, treatment

## Abstract

**Background:**

Opioids are one of the most prescribed treatments for chronic pain (CP). However, their long‐term use (>3 months) has been surrounded by controversy, due to loss of beneficial effects.

**Objective:**

To explore the experiences of people with chronic non‐malignant low back pain in Spain undergoing long‐term treatment with opioids.

**Design:**

Qualitative study.

**Setting and participants:**

We conducted 15 semi‐structured interviews at the Pain Clinic with persons taking opioid treatment.

**Methods:**

The interviews were analysed by qualitative content analysis as described by Graneheim and Lundman, and developed categories and themes discussed in light of a biomedicalization framework.

**Main results:**

We developed one overarching theme—*Living with opioids: dependence and autonomy while seeking relief*—and three categories: *The long pathway to opioids due to the invisibility of pain; Opioids: from blind date to a long‐term relationship*; and *What opioids cannot fix*.

**Discussion:**

The long and difficult road to find effective treatments was a fundamental part of coping with pain, involving long‐term relationships with the health system. This study reflects the benefits, and drawbacks of opioids, along with struggles to maintain autonomy and make decisions while undergoing long‐term treatment with opioids. The paper also highlights the consequences of pain in the economy, family and social life of patients.

**Conclusions:**

Patients' experiences should be considered to a greater extent by health‐care professionals when giving information about opioids and setting treatment goals. Greater consideration of the social determinants of health that affect CP experiences might lead to more effective solutions to CP.

## 1 INTRODUCTION

Chronic pain (CP), defined as pain that persists beyond the normal tissue healing time (3 months as a convenient cut‐off point), is a health problem that has reached epidemic proportions worldwide. The average CP prevalence is 27% in European countries, consistent with international estimates.^1^ In Spain, around 17% of the population suffer from this illness, making it a major health‐care problem.^2^

Opioids are one of the most prescribed analgesic pharmacological treatments for CP.^3^ Prescriptions for opioids have increased dramatically in the last few decades, numbers being far higher in countries such as the United States or Canada.^4^ According to the National American Survey on Drug Use and Health (NSDUH) report in 2016, more than one‐third of American adults were prescribed opioids.^5^ Although to a lesser extent, an increase has also been observed in some European countries,^6^, ^7^, ^8^ including Spain (83.59% from 2008 to 2015).^9^

Opioid therapy has been found to be associated with the alleviation of pain in the short term.^10^ However, their prescription for long‐term use sometimes presents a dilemma,^11^ since it has been accompanied by a great increase in overdoses, abuse, addiction and recreational use in some countries such as the United States.^12^ Clinicians therefore face the potentially conflicting duties of relieving pain on the one hand, viewed worldwide as an ethical medical obligation,^13^ and preventing the potential harm to the patient of long‐term opioid consumption on the other.^14^ Dispelling the myriad myths associated with pain and fears associated with opioid prescription is no easy matter. Part of the solution depends on educating and training health‐care professionals in pain management.^15^

Patients who experience CP could also face this quandary since opioids have been related to negative side effects, such as excessive sedation, respiratory failure, urinary retention or constipation.^16^ These side effects, along with social, cultural and historical factors, have given rise to a set of attitudes and beliefs regarding the deleterious effects of opioid administration for pain relief.^17^ The main reasons for this inappropriate set of attitudes and beliefs are the lack of knowledge regarding opioids and the stigmatization that some patients felt when prescribed opioids.^18^

The benefits of opioids have also been surrounded by controversy.^19^ Some studies^20^, ^21^ have shown that most people using opioids continue to report moderate or severe pain, and that functional improvements are often limited. Other authors^22^ have shown that increases in the intensity of pain are connected with skipping doses, since patients reduce or stop taking their opioid therapy to avoid side effects. Despite the existence of clinical guidelines that regulate the correct use of opioids in the treatment of pain,^23^, ^24^ in Spain, CP management still remains weak.^25^ Fear of addiction might be an important reason why CP is often undertreated.^16^

Recent literature^18^, ^26^, ^27^, ^28^ has explored the experience of adults using prescription opioids to manage CNCP, concluding that there were many negative aspects to using opioids daily, in most cases these were outweighed by the positive effects, and most of the negative aspects were socioculturally induced rather than caused by the drug itself. However, these studies have been carried out in countries such as the United States or Canada, where the trend of opioid use has been accompanied by an increase in reported opioid abuse and opioid‐related death^29^ which is a different situation compared to Spain.^30^, ^31^, ^32^, ^33^

Considering the potential worries and difficulties associated with the use of opioids, it is very important that patients communicate and relate to health‐care providers their experience with opioids in an open and effective manner. However, although the number of individuals living with CP and taking opioids is increasing, to the best of our knowledge, no study has been performed to explore the experiences of patients taking opioid medications in Spain. Thus, this study aims to fill this gap in knowledge by exploring the experiences of patients with CP receiving long‐term treatment (more than 3 months) with opioids in Spain.

### 1.1 Spanish health‐care system

The Spanish health‐care system has universal coverage, is almost entirely funded by taxes and is free of charge at the point of delivery, except for pharmaceutical products for people under 65, which require a copayment of 40% of their price. Provision of care is predominantly within the public network of health‐care facilities. Primary health care is the first point of contact for individuals with the health‐care system, and thus, professionals working there as GPs, nurses and midwifes act as gatekeepers of the system. The primary health‐care network is an integrated part of the public systems through mutually supportive referral systems with secondary and tertiary health‐care facilities. In the case of patients who experience CP, after visiting their GP they are referred to a specialist, usually a rheumatologist or traumatologist, and then to the Pain Clinic if the pain does not remit.

The Pain Clinic is a unit specialized in the management and treatment of all complex types of pain conditions, especially in those patients who do not respond to conventional treatment and those who require special drugs or treatment techniques, such as local infiltration of anaesthetics and/or steroids or radiofrequency neurolysis.

Opioid treatments can be prescribed by GPs or specialist doctors. All official opioid prescriptions must include the denomination ‘Official Narcotics Prescription’, with the exception of those that are issued in electronic format. In each Official Narcotics Prescription, only one type of opioid treatment must be prescribed, with a maximum treatment length of 3 months and without exceeding a total of four containers.

### 1.2 Theoretical framework

CP is considered to be a complex biopsychosocial event.^34^ Besides the physical experience of pain, individuals suffering from CP often experience mental and emotional disturbances and their family environment might also be severely affected.^35^ Given its complexity, health‐care services should address CP following a multidisciplinary approach, although pharmacological therapy is still considered the cornerstone (and sometimes the only approach) of the control of pain.^36^ Moreover, as we have previously described, long‐term treatment with opioids might help to relieve pain in some cases, but result in other issues related not only with adverse effects, but also with communication, negotiation and power relationship problems between patients and providers, stigma and the role of family and support networks. Following an emergent design, a biomedicalization framework was chosen, meaning that the analysis of the interviews guided the choice of theory. Thus, the theoretical framework was mainly used in the discussion section to contrast the results of this study with previous evidence and to frame participants' experiences in the wider context of health care. Biomedicalization was described by Clarke et al (2003) as the ‘increasingly complex, multi‐sited, multidirectional processes of medicalization, both extended and reconstituted through the new social forms of highly technoscientific biomedicine’. Biomedicalization is driven by and at the same time fosters five key overlapping processes: major shifts in health and health‐care policies and funding; the focus on health itself and elaboration of risk and surveillance biomedicines; technoscientization of biomedicine; major changes in the production and consumption of biomedical knowledge; and transformation of bodies and new individual and collective identities. The results of this study are better understood and explained in the light of this framework as we will explain in the Discussion section.

## 2 MATERIAL AND METHODS

### 2.1 Study design

This is a qualitative study in which data were collected through 15 semi‐structured interviews to explore the experiences of chronic low back pain (CLBP) patients taking opioids to treat their pain. Individual interviews were analysed by qualitative content analysis as applied in health sciences research.^37^

### 2.2 Participants and data collection

The study protocol was approved by the Clinical Research Ethics Committee of the ‘Puerta del Mar’ University Hospital (Cádiz, Spain), ensuring compliance with the standards of good clinical practice.

Recruitment and data collection were conducted from April to October 2018. The participants were recruited from the Pain Clinic in Hospital Puerta del Mar. Inclusion criteria for the study were as follows: adults suffering from chronic non‐malignant low back pain and receiving long‐term treatment (over 3 months) with opioids. Patients taking opioids for less than 3 months or with another pain origin than chronic non‐cancer low back pain were not included.

All the patients were recruited after a routine physical evaluation in their medical visit to the Pain Clinic. Previously, their medical data, including information on prescribed medications from the records, were evaluated and discussed by the clinician and interviewer. If the person met the inclusion criteria after an analysis of their medical records and their medical visit and physical evaluation, the clinician explained to him or her the aim of the study. All seventeen eligible patients were approached by the clinician. After this initial approach by the clinician, the interviewer met the potential participant and they went to a quieter place in a clinical setting for the interview, before with the participant was shown a letter with more comprehensive information about the study and its aim. The participants were left alone to read and think carefully before giving their written informed consent. When they finished reading it, they had the opportunity to ask questions about the study, after which the interview took place. At this stage, two people rejected the participation, alluding to lack of time. Individual, semi‐structured, qualitative interviews following a guide were conducted in Spanish. The guide was based on open‐ended questions developed with guidance from the literature regarding chronic pain experiences and factors associated with the use of opioids (Table 1). Aspects related to the origin of their pain, opioid belief, information received about treatment, opioid experience, their family and social support were also of particular interest. If a specific topic that was not included in the first version of the interview guide came to light spontaneously in a specific interview, it was added and asked in the subsequent interviews. Interviews were audio‐recorded, transcribed verbatim and anonymized. All names used here are pseudonyms. We conducted interviews until very similar experiences were described in the last interviews as in the previous interviews.

**TABLE 1:** Interview guide used for the semi‐structured interviews

Exploring pain
How did the pain start?
Exploring prescription of opioids and information
Can you tell me about how you started taking opioids?
How did your doctor suggest taking these medications?
How did you react when your doctor told you that you will be taking an opioid treatment?
What was your opinion about opioid medications before taking it? And now? Has your opinion changed?
Were other alternatives considered? Which ones?
What type of information about opioids have you received?
What is your opinion about the information that your doctor gave you about the treatment?
Have you sought information through other means? Which ones?
Daily life and opioids
Can you describe a usual day in your life?
Since you started this treatment with opioids, have you changed your daily activities?
Since you started this treatment with opioids, could you describe how your health is?
Could you describe your mood since you started this treatment with opioids?
Do you think that opioids cause side effects? How?
Taking opioids
Have you ever tried to stop or decrease the opioid dose? Why? How was the experience?
Since you started this treatment, have you needed to increase the dose? Do you think you would have needed to increase the doses? Why?
What is the best way to relieve your pain?
Social relationships and work
How is your social life since you are under an opioid treatment?
Can you describe the relationship with your family? Has this relationship changed since you are under treatment?
Can you describe your working life?
Final questions
How is your experience with opioid treatments In general?
Would you like to add anything else?

### 2.3 Analysis

We adopted a constructionist perspective. We analysed all the interview transcripts following qualitative content analysis as described by Graneheim and Lundman.^37^ The data analysis was inductive, and thus, the category construction was data‐driven; no initial hypothesis guided the preliminary coding and subsequent development of categories. However, in the analysis of the results presented in the Discussion section, we followed the biomedicalization framework described above.

Interview transcripts were entered into Atlas.ti 1.0.16 to support the coding process. At the beginning of each interview transcript, a brief log of the interview was written, including information about the time, duration, and the feelings and perceptions of the interviewer during the conversation in order to help with the analysis process. The researcher who conducted the interviews transcribed them verbatim.

To carry out the qualitative content analysis, two researchers read the transcripts independently and assigned codes line‐by‐line to meaningful pieces of the interview transcripts. Then, the researchers met to compare and refine codes, which were then grouped into categories. The material was grouped into three key categories, which were further validated after re‐analysis of all the interviews. Coding maps were used to help with the code grouping and the analysis of relationships between the emerging categories and codes. In the last step, an overarching theme involving these three categories was identified. The analysis was conducted in Spanish, and quotes were chosen from this material to be translated into English. All the authors understand both languages and, thus, were able to participate in the whole analysis process.

Our positions as researchers have continuously been discussed in relation to ethical considerations and questions about responsibility. In line with Graneheim and Lundman (2004),^37^ we argue that, in qualitative content analysis, interpretation involves a balancing act of providing interpretation while at the same time making sure that our interpretations remain always grounded on the data. By providing a thorough explanation of the analytical process, our intention is to allow the reader to assess the study's usefulness and transferability.

## 3 RESULTS

Fifteen people aged from 40 to 88 were interviewed (9 women and 6 men). One participant had completed higher education and the rest elementary education. Four had a declaration of total disability to work, two were on sick leave, and nine were retired or unemployed. Thirteen were prescribed a treatment with a strong opioid, one of the two who were taking weak opioids had a PRN order (Table 2 near here).

**TABLE 2:** Characteristics of the sample

	Gender	Age	Income/Occupation	Years with pain	Under opioids' treatment	Opioid currently prescribed
Julia	Female	88	Retirement (Housewife)	15	1 y	Tapentadol
María	Female	40	Total permanent disability (Administrative assistant)	22	8 y	Tapentadol
Laura	Female	55	Sick leave (Administrative assistant)	14	1 y	Tramadol (PRN)
Juan	Male	72	Retirement (Accountant)	4	4 y	Tapentadol
Pilar	Female	56	Unemployed (Housewife)	13	13 y	Tapentadol
Lola	Female	41	Total permanent disability (Administrative assistant)	8	8 y	Tapentadol
Carlos	Male	66	Retirement (Bricklayer)	2	6 mo	Tapentadol
Rafael	Male	52	Sick leave (Watchman)	5	5 y	Tapentadol
Ana	Female	41	Unemployed (Housewife)	7	5 y	Tramadol
Leticia	Female	72	Retirement (Hairdresser)	48	4 y	Tramadol y Tapentadol
Sofia	Female	46	Total permanent disability (Cleaner)	2	2 y	Oxycodone
Alejandro	Male	51	Total permanent disability (Bricklayer)	9	4 y	Morphine
Roberto	Male	72	Retirement (Mechanic)	“Long time ago”	2 y	Tapentadol
Carmen	Female	80	Retirement (Cleaner)	30	“Many years”	Tramadol
Hugo	Male	52	Unemployed (Carpenter)	5	5 y	Morphine

From the analysis, one overarching theme was developed: ‘Living with opioids: dependence and autonomy while seeking relief’, which crosscut three categories: ‘The long pathway to opioids due to the invisibility of pain’; ‘opioids: from blind date to a long‐term relationship’; and ‘what opioids cannot fix’.

The quest for effective treatment was a fundamental part of the participants' struggle to cope with the pain, and it involved long‐term relationships with the health system. In relation to this, the theme ‘living with opioids: dependence and autonomy while seeking relief’ refers to how navigating the health system meant that the study participants were dependent on health‐care professionals exercising their power to refer them to specialized care to get access to a diagnosis and treatment, including opioids. At the same time, it also meant having, to a certain extent, room to make decisions, to exercise autonomy, despite having little information and meeting professionals that hardly coordinated/communicated with each other.

The two first categories ‘the long pathway to opioids due to the invisibility of pain’ and ‘opioids: from blind date to a long‐term relationship’ refer to the journey participants made to get a diagnosis and treatment with opioids, and their experiences during this long and difficult process, which was quite unique for each person. The third category, ‘what opioids cannot fix’, describes the circumstances and situations experienced by the patients before and after the painful episode started, and how they have influenced the whole process. In this case, opioids do not have any effect since they are not enough to remedy the deficiencies derived from these situations.

### 3.1 The long pathway to opioids due to the invisibility of pain

This first category describes the long and difficult pathway that participants followed from the onset of their pain until getting treatment with opioids. This journey could start as soon as early adolescence (Table 3).

**TABLE 3:** Quotations illustrating categories and theme

Theme: “Living with opioids: dependence and autonomy while seeking relief”
**Category: “The long pathway to opioids due to the invisibility of pain”** **Ana, 41 years old, 5 years taking tramadol:** “since I was a kid, I've always had back pain. I had to come frequently to the hospital, always with “lumbago”, mainly “lumbago”. **Carlos, 66 years old, 6 months tapentadol**: I have had this problem for at least 2 years, and it has been getting worse and worse… I wanted the doctors to see me, but there was a long waiting list for the specialist. I had to go to the emergency room because I couldn't take it anymore. Over and over again to the emergency room… and again a dropper, only a dropper… until finally the neurosurgeon saw me and said: something must be done here, and he sent me here to the Pain Clinic. **Rafael, 52 years old, 5 years taking tapentadol:** (talking about a previous health problem in the eye) “well, it was more visible, the eye was really red, red, red, and people asked me “What happened? Did you hit something or what did you do?” […] You could see the surprised and shocked faces. But now (with Chronic Pain (CP))… they see me so fine, so healthy, and in my inside, I am dying with pain”. **Lola, 41 years old, 8 years taking tapentadol:** “My parents took me first to the emergency room, and they injected me with morphine, and sent me home in an ambulance […] The following day it was the same. Another injection and go home. Then, they told me that I needed surgery because all my lumbar area was calcified. Then I could feel something strange… and they prescribed (small pause) morphine, and each time the dosage was increased more, and more… and there was no pain relief. They told me there were two possible big issues with my type of surgery: instability remaining in the backbone or leaving residues of spinal discs, and I had both”.
**Category: “Opioids: from blind date to a long‐term relationship”** **Laura, 55 years old, 1 year taking tramadol:** “The doctor told me: ‘I'm going to prescribe you this medication that I think is going to help you’. I found out later that tramadol was an opioid, when I searched it on the internet, and people told me: ‘uh, that medicine has this and that…’ and I said, ‘that much?’ ‘yes, yes, it's like morphine, a derivative’. After this, I saw what it was. But my doctor didn't tell me anything.” **Sofia, 46 years old, 2 years taking oxycodone:** “I didn't know what morphine was. I had heard that it was for drug addicts, that's the truth… I didn't know… That's what I have always heard in my house. I knew there were patches and all that stuff, but I didn't know for what exactly. **Lola, 41 years old, 8 years taking tapentadol:** “I was very wrong […] I remember that morphine was used with my grandfather before dying to relieve his pain and all that stuff, so I never thought I would have to take it as they say “as an outpatient treatment, at home.” I didn't think it would be like that”. **María, 8 years old, 8 years taking tapentadol:** “The morphine has caused me… I sleep with a CPAP because of the dryness that morphine left me. It caused me sleep apnea. It's left many side‐effects apart from my illness because all the medication that I have been taken. **Ana, 41 years old, 5 years taking tramadol**: “It's true that opioids must have some effect, because I remember that once I ran out of them… and I do not know if it was a day or two without taking them, and look, I was a physical wreck. I had to lie in bed […] When I bought and took them, it made me “Boom!”. I rush out to the shops! My husband was shocked! I said: “the pill, look what the pill has done to me”.
**Category: “What opioids cannot fix”** **Ana, 41 years old, 5 years taking tramadol:** “For some time I had to take anxiolytics. The doctor said that my problem had no solution, that I was going to be in pain forever, and I fell apart. But it's true that I had no other option. I always try to say to myself: ‘no, come on, I'm not going to cry’. This is what I have and that's all there is to it. Your mind is the one that has to say: ‘hey, we stop here, I will not cry’. **Pilar, 56 years old, 8 years taking tapentadol**: “I have little help to be honest. I often get angry with him [her husband] because of that. All the work is for me. Well, if I'm cleaning, of course he helps me, but the housework… he doesn't… he hangs out the washing, he helps me with little things like that, he helps me making the bed and that's all. **María, 8 years old, 8 years taking tapentadol:** “I speak to my very close friends and I have to explain that it's morphine and that this is what I need for my chronic pain. The society is not… not well informed; it's seen like… phew… so bad, she's dying or it must be bad if she is taking morphine, and it's not like that”. **Lola, 41 years old, 8 years taking tapentadol:** “My family has been my lifesaver. Without my father and my mother… look, I am giving more importance to them than to my partner. During the day, my partner is not with me. He cannot stop working to stay with me… it is understandable with just one salary for both of us. If he stops working, we cannot live.”

For the participants, the fact that pain ‘cannot be seen’ explained such a lengthy journey to obtain diagnosis and prescriptions. They mentioned numerous consequences of this invisibility of pain in individual and social spheres, as well as in their encounters with the health‐care system. At the individual level, the invisibility of pain meant that it could be ignored or minimized by those suffering from it. As Alejandro (51 years old, 4 years of taking morphine) said, ‘mine [pain] was caused by work, by lifting weight, my back started hurting, and… I was walking and limping, and I thought it was…well, nothing. I thought it will go away. By the time I realized and went to the doctor, I was using crutches’.

In the social arena, the long history of pain, together with the lack of physical signs, could lead, in the view of participants, to indifference or a lack of empathy. As Hugo (52 years old, 5 years of taking morphine) claimed, ‘my family say that I'm exaggerating’. Relatives and friends were described as having got used to seeing participants in pain and therefore minimized its importance. Participants described the difficulty in lending credibility to the severity of the problem when there were no physical signs. As Rafael (52 years old, 5 years of taking tapentadol) put it: ‘They've seen me in pain for so long… I think ‘if they could know how much pain I feel' but they see me every day in the same situation and they've become used to seeing me in pain’.

In relation to the health‐care services, participants described how they had to struggle with health‐care professionals to be believed and have their pain taken seriously, as Laura referred (Table 3). Similar to what happened in the individual sphere, referrals from primary and emergency care to specialized pain services did not begin until the patient's mobility was severely affected, until the pain manifested itself through physical signs or until they visited the same facility several times without improvement. That led to long waiting times and delays in receiving an appropriate diagnosis and treatment.

Entering the referral system was the beginning of a tortuous journey of hopes and disappointments, a trial/error process that involved trying different treatments with the dream of a pain‐free life, as the quote from Lola portrays (Table 3).

The Pain Clinic was commonly the place where the long‐term treatment with opioids was established, although in some cases treatment with opioids had started before reaching such specialized services, that is in primary health‐care facilities. This relationship with opioids, frequently initiated at the Pain Clinic, is the focus of the next category.

### 3.2 Opioids: from blind date to a long‐term relationship

This category portrays beliefs and perceptions of the benefits and drawbacks of treatment with opioids, in addition to struggles to keep autonomy and make decisions while being a long‐term patient within the health system.

It was difficult for participants to recall their first contact with opioids, since the common experience of the interviewees was that they had been given little or no information about the new medication they were prescribed. Consequently, it was difficult for participants to distinguish between medications that were in fact opioids and other drugs (Table 3).

When the participants realized that they were being prescribed opioids, they seemed to accept the treatment due to the intensity of pain suffered, despite having the perception that opioids were for terminal diseases or relating them with drugs and addiction (Table 3, Sofia and Lola quotations). Yet, the perception of opioids as a ‘serious’ prescription was maintained over time and the fact that their acquisition is regulated and controlled was mentioned repeatedly in the interviews. There is also a paradox since although some of the participants noticed adverse effects of their medication and they reflected on the difficulty involved in quitting this long‐term treatment they weighed in favour of relief. As Sofia (46 years old, 2 years of taking oxycodone) said: ‘The truth is that they benefited me, I mean, I experienced no strange reactions… well, drowsiness, I am like an animal in hibernation, sleeping the whole day’. This understating of the effects—drowsiness, in that case—could also be related to the lack of information received from health‐care professionals. As we see in the next quote, Rafael explained commonly experienced adverse effects with opioids like tolerance and dependence without naming them specifically:

The point is that coincidentally my illness has become worse, and thus, they go parallelly, the increase in medication dosage and the increase in pain, and consequently they are increasing the dosage because my pain is getting worse. The pain I had six months ago is now worse. What's happening is it's like my body got used to the treatment.

As time passed, the participants appeared to start taking a more active attitude towards coping with pain. They described how they had learned ways to relieve the pain, including resting, losing some weight, exercising (eg swimming, Pilates, walking) and taking other medication as needed (eg muscle relaxants, non‐steroidal anti‐inflammatory drugs). As a reaction to the adverse effects experienced, they seemed to become progressively more active in decision‐making related to pain management, and less likely to rely exclusively on opioids. In this sense, medication‐related decisions were frequently made without consulting the health‐care professionals.

It was the bad sweating that I suffered… I read the information pamphlet and read sweating was an adverse effect and then I wondered, ‘what if I reduce a little bit the dose? Let's do an experiment!’ I thought ‘maybe the doctor will get angry with me, but I am going to experiment’, without quitting totally. I thought “I am going to take less than what I was told, and I'll see if I can continue without pain and avoid that unpleasant sweating” and, right now, indeed, I am taking half of the pill. Pilar (56 years old, 8 years of taking tapentadol)

But these more active coping strategies to reduce pain did not mean total scepticism of opioids. Although the participants complained that opioids had not totally eliminated the pain, there was a common feeling that they contributed to pain reduction. Sofía said: ‘I was unable to take a step, and thanks to starting to take morphine, I can now stand up. If I was not taking it, I wouldn't be doing what I am; I do minimal things but I at least do them’.

The interviews unveiled that their experience with opioids was strongly intertwined with many other life circumstances that lie far beyond the scope of action of any medication, as explained in the next category.

### 3.3 What opioids cannot fix

This category describes different spheres of the participants' lives where pain has an impact on a range of economic, familiar and/or social issues that cannot be addressed through opioids (alone). Moreover, these issues may not only be the consequence of pain, but what caused it in the first place.

Besides physical limitations and problems, the emotional sphere was one of the most strongly affected areas, one that opioids could not improve and could even affect negatively. Although in some cases reductions in pain led to a better mood, in others, sadness due to physical limitations and fear of pain was constant. The next quote reflects how pain (and the opioid medication to treat it) had disrupted a participant's life and hindered them from doing basic daily activities.

I have noticed changes in my mood, you know? I have …. a strong personality. I don't know whether it's the pill or whether it's the…. Not being able to move as I wish, not being able to do things the way I would like to. I often feel useless, even with my partner… I cannot even have sex as a normal person would, I'm limited! Laura (55 years old, 1 year of taking tramadol)

In certain cases, these decreases in mood caused by pain resulted in mental health comorbidities among the participants. Sometimes this was exacerbated when participants were told about the chronicity of their illness (Table 3).

To navigate life suffering pain, family support was regarded as essential by the participants. However, at the same time, being dependent on their help because of their physical limitations raised perceptions of being a burden. Moreover, as described in the first category, participants related sometimes feeling neglected, as if their families had got used to seeing them in pain. Roberto (72 years old, 2 years of taking tapentadol) explained: ‘They help me, everything that needs to be done now it's done by my sons‐in‐law, poor them, because I can't. But you often feel useless; it bothers you that someone is working hard on your behalf, but they are very nice’.”

The women participating in the study, regardless of whether they were on sick leave or retired, did not identify housework and childcare as work, and they mentioned still being the only person responsible for housework despite their disabling pain. In addition, as the Pilar quote shows in Table 3, women refer to the ‘little things’ that their partners do at home as their ‘help’, showing that housework is not a shared responsibility from the start.

The participants were aware of the importance of maintaining an active social life, and consequently appeared to make an effort to do so. This was easier with people who shared the same pathology, problems and treatment. Lola (41 years old, 8 years of taking tapentadol) said: ‘I'm part of a Facebook group of people with the same surgery as I had. You can hear some people encouraging others; at night you know that half of them have been awake like you, and you are there… and that cheers you up a little bit. Now, I'm surprised to see how many people are taking opioids; indeed, we all take them as if it was water. It's much more common than I had ever imagined’. Outside ‘pain friends’ circles, the experience was different and participants related having felt judged by other people when they disclosed they were taking opioids.

Narratives of hard lives where pain was ‘just’ another added difficulty were a constant in the interviews. Having performed manual labour from a young age was common among these patients, and sometimes the cause of the illness and the pain. Sofía stated: ‘I was a cleaner, and they told me ‘throw this in the garbage’ and I pulled the trolley and that was it… because my back creaked and afterwards my back was destroyed from my job as a cleaning woman’. Likewise, poor working conditions and living with economic difficulties appeared intertwined as a cause and consequence of pain. As Rafael mentioned: ‘At home the only income is my salary. I have to pay the mortgage, for my children's studies… well, our income is reduced as I'm on sick leave…so I cannot stop working and this situation has led me to a state of anxiety’. As is the case with family support, there were also differences between men and women related to economic difficulties. For two men who participated in the study, the pressure of being the breadwinner had negative emotional consequences. For three of the women, being economically dependent on their partners led to them having feelings of helplessness. Ana (41 years old, 5 years of taking tramadol) said: ‘The point is that I get on well with my husband, but if I did not get on well with him… what could I do? I'm unemployed, I don't have anything, (she gets emotional) ‘my God’.

## 4 DISCUSSION

The findings of this study show how the experience of relieving pain is a constant struggle among people who suffer from it. Opioids become a way of reducing pain, facilitating physical and social functioning and making a more independent lifestyle possible. However, these feelings of independence about physical or social functioning are in conflict with concerns of dependence on the medication.

Our results described how the participants' experiences were severely influenced by the invisibility of their pain, in the same line as findings from previous research.^38^, ^39^, ^40^ In this sense, the absence of a uniform classification and a validated diagnostic tool in this type of pathology hinders the standardization of treatments that biomedicalization has brought to other diseases, leading to uncertainty in the treatment and diagnosis of the patient, and making it very much dependent on the individual perception of the treating physician.^40^, ^41^ Thus, as we have seen in our results, the choice of where to refer patients and how to treat them is a lengthy trial and error process, which is certainly not ideal, and opens the door to disparate access to health care.

In this study, the overwhelming majority of participants eventually treated in the Pain Clinic after lengthy periods navigating through the health‐care system were employed in unskilled jobs and reported having a basic level of education. We argue, based on the discourse of biomedicalization,^42^ that this overrepresentation of patients from lower socio‐economic backgrounds in our study is because the quest for a diagnosis and treatment could presumably be ‘easier’ and ‘shorter’ for those who can afford private medical care and can skip the mutual referral system between the GPs and specialists of the public health system. The increase in stratifying fee‐for‐service options, which is another characteristic of biomedicalization, enables the more wealthy to address their illness, circumventing waiting times for medical procedures and obtaining access to multidisciplinary intervention in the biological, psychological and social aspects of their chronic pain condition.^43^

In relation to experiences with opioids, our findings show that opioids were insufficient to relieve all the pain, as examples of limitations to daily life because of pain were an important part of the participants' narratives. However, the participants' perception was also that their physical functioning and quality of life had improved thanks to opioids. These contradictions between perceived improvement and narratives of severe limitations were recurrent in the participants' accounts. As other authors have shown,^18^ due to a lack of information or misinformation, patient expectations regarding the results they can expect from this treatment may be unrealistic. In line with this, the difficulties the participants found to identify side effects, tolerance or dependence were also noteworthy. Exemplifying the heterogeneity in the production, distribution and access to biomedical knowledge that is part of biomedicalization, the majority of the participants stated that they had looked on the Internet to be informed about opioids and their consequences, and in many cases developed individual strategies to deal with the side effects they experienced. Having to look oneself for information about the treatment prescribed reflects a shift in responsibility for care practices, which is put increasingly on healthcare‐users, this change being another essential component of biomedicalization.^42^ Even if access to medical knowledge is improved thanks to new technologies, the ability to benefit from that information depends strongly on individual health literacy levels. According to a report published by the WHO in 2013,^44^ more than half of the Spanish population have inadequate or problematic health literacy levels.^44^ Furthermore, the population with a lower social status have much higher proportions of limited health literacy levels.^45^, ^46^ In addition, increased responsibility for their own healthcare driven by biomedicalization processes leads to self‐blame for any health problems that arise. As seen in some of the quotations of the participants, they blamed their pain on things they have done, like pulling too much weight or waiting too long to seek medical care instead of placing the blame on their working conditions when evidence shows that unskilled workers have the highest prevalence of musculoskeletal disorders, a large proportion of which can certainly be attributed to working conditions.^47^

Finally, another basic process of biomedicalization is the production of new identities and reframing of old ones by technoscientific means. In this sense, the social role and identity of chronic patients for whom no cure is available despite all the technoscientific advances remain a challenge due to the invisibility of pain. In the social arena, the participants of our study felt stigmatized in several ways. They felt neglected due to the invisibility of pain. Many patients with CP do not present any visible symptoms and remain stoic when they feel pain, resulting in a lack of empathy from friends.^48^ In addition, the participants felt stigmatized because of the treatment with opioids. Ljungvall et al^26^ described how participants experienced being stigmatized because of their repeated contacts with medical care workers, who see them as drug‐seeking behaviours. However, in our study, the participants experienced this stigmatization by relatives and friends who expressed many concerns and prejudices about opioids. The negative consequences of ‘double’ or ‘layered’ stigmatized conditions—‘CP sufferer’ and ‘drug addict’—have also been described by Dassieu et al,^31^ who suggest that being doubly stigmatized reinforces people's isolation as well as their experience of loss of dignity. Interestingly, in our study, the participants referred to sharing their experience of CP and opioid treatments with ‘pain friends’, among whom treatment with opioids was common. Peer support has been shown^49^ to reduce social isolation, encourage shared experiential learning and foster psychosocial well‐being. Thus, ‘taking charge’ of their health, understood in biomedicalization as responsibility for care practices, by means of active coping strategies such as maintaining an active social life with ‘pain friends’ was crucial for the health of the participants, as reported in the literature.^18^

As previously described,^28^, ^50^ family support was also very important for the participants with regard to the management of their pain. Some, however, considered themselves to be a burden due to their physical disability. This feeling could result from a sense of inequity or imbalance if they perceive that what they receive outweighs what they provide.^51^ In addition, some participants described feelings of depression and uncertainty, as well as a decreased sense of autonomy and/or self‐confidence because of both physical dependence and economic dependence. These worries emerge strongly in this study. As a result, physicians often find themselves trying to bridge the gap between the chronic pain patient's expectations for effective pain relief and the harsh biomedical reality.^52^ However, medication, opioids in these cases, is not enough to solve problems of another nature that in some cases predated the pain.

## 5 CLINICAL IMPLICATIONS

Greater awareness should be raised among health‐care professionals about the experiences of patients with CP to counterbalance the negative effects of the invisibility of pain such as the lack of credibility given to symptoms expressed by patients and delays in access to diagnosis and treatment. This would shorten and improve patients' relationship with health‐care services.

Regarding information about long‐term treatment with opioids, there is a need to improve the quantity and quality of information provided to patients. Furthermore, health‐care professionals should be aware of the extended use of alternative information sources like Internet or peers among patients who experience CP, to ensure they are able to assess the credibility of the information they access and are able to understand and make use of this information, that is to ensure they have sufficient health literacy levels.

Finally, an interdisciplinary approach and health‐care team that includes social workers and psychologists is essential to address all those spheres of life affected by and affecting CP experiences and where opioids have little or no effect.

## 6 METHODOLOGICAL CONSIDERATIONS

As previously described, several steps were taken to strengthen the trustworthiness of the findings. These do, however, need to be interpreted with some limitations in mind. Concerning transferability, it is important to consider the context where this study was conducted: a group of individuals with chronic pain, treated in a Pain Clinic of the Spanish health‐care system. With this in mind, we consider that the results from this study could be relevant for understanding the experiences of people with CP who are taking long‐term treatment with opioids in other countries with similar sociocultural aspects and health‐care systems, since the consequences they face and concerns they have about opioids may be the same.

Regarding credibility, we chose participants with different sex/gender, ages and experiences to increase the likelihood of shedding light on the research question. What is more, the open‐ended questions made it possible to share both positive and negative experiences. However, people who were at the beginning of the illness process may not have been reached, since we recruited participants via the Pain Clinic. Nonetheless, as we discuss in this study, CP is an illness that implies a long and difficult process before being be diagnosed and treated. Thus, opioids as a treatment are usually prescribed to those who have been suffering pain for a long‐term period.

Another limitation of this study is that, although the results suggested gender differences in the patients' experiences with both CP and its treatment with opioids, the data were not rich enough to support a deep analysis and the elaboration of conclusions. Further research with this aim is required.

## 7 CONCLUSIONS

The participants' experiences were strongly shaped by the invisibility of pain, which led to a long‐term relationship with the health‐care system and different forms of stigmatization. The participants made up for the limited information received from health‐care professionals by surfing the Internet or asking peers. Yet, they showed limited knowledge about side effects and the long‐term consequences of the treatment.

The burden of social determinants of health was increased by CP and at the same time a source of complications in CP experiences.

## CONFLICT OF INTEREST

No potential conflict of interest was reported by the authors.

## ETHICAL APPROVAL

The study protocol was approved by the Clinical Research Ethics Committee of the ‘Puerta del Mar’ University Hospital (Cádiz, Spain), ensuring compliance with the standards of good clinical practice.

## Data Availability

The data that support the findings of this study are available on request from the corresponding author. The data are not publicly available due to privacy or ethical restrictions.

## References

[1] Lin J , Scott W , Carpenter L , et al. Acceptance and commitment therapy for chronic pain: protocol of a systematic review and individual participant data meta‐analysis. Syst Rev. 2019;8(1):1‐10. 31200768 10.1186/s13643-019-1044-2 PMC6570828

[2] Dueñas M , Salazar A , Ojeda B , et al. A nationwide study of chronic pain prevalence in the general Spanish population: identifying clinical subgroups through cluster analysis. Pain Med. 2015;16(4):811‐822. 25530229 10.1111/pme.12640

[3] Dowell D , Haegerich TM , Chou R . CDC guideline for prescribing opioids for chronic pain — United States, 2016. JAMA. 2016;315(15):1624‐1645. 26977696 10.1001/jama.2016.1464 PMC6390846

[4] Fischer B , Jones W , Rehm J . Trends and changes in prescription opioid analgesic dispensing in Canada 2005–2012: an update with a focus on recent interventions. BMC Health Serv Res. 2014;14:1‐8. 24572005 10.1186/1472-6963-14-90 PMC3941687

[5] Walker G . The opioid crisis: a 21st century pain. Drugs Today. 2018;54(4):283‐286. 10.1358/dot.2018.54.4.2812620 29869649

[6] Birke H , Kurita GP , Sjøgren P , et al. Chronic non‐cancer pain and the epidemic prescription of opioids in the Danish population: trends from 2000 to 2013. Acta Anaesthesiol Scand. 2016;60(5):623‐633. 26861026 10.1111/aas.12700

[7] Werber A , Marschall U , Helmut L , Hauser W , Moradi B , Schiltenwolf M . Opioid therapy in the treatment of chronic pain conditions in Germany. Pain Physician. 2015;18:E323‐E332. 26000679

[8] Todd A , Akhter N , Cairns J‐MM , et al. The pain divide: a cross‐sectional analysis of chronic pain prevalence, pain intensity and opioid utilisation in England. BMJ Open. 2018;8(7):1‐9. 10.1136/bmjopen-2018-023391 PMC6144392 30206064

[9] Agencia Española de Medicamentos y Productos Sanitarios . Utilización de Medicamentos Opioides En España Durante El Periodo 2008‐2015; 2017 https://www.aemps.gob.es/medicamentosUsoHumano/observatorio/docs/opioides‐2008‐2015.pdf

[10] Kalso E , Edwards JE , Moore RA , McQuay HJ . Opioids in chronic non‐cancer pain: systematic review of efficacy and safety. Pain. 2004;112(3):372‐380. 15561393 10.1016/j.pain.2004.09.019

[11] Carrico JA , Mahoney K , Raymond KM , et al. The association of patient satisfaction‐based incentives with primary care physician opioid prescribing. J Am Board Fam Med. 2018;31(6):941‐943. 30413550 10.3122/jabfm.2018.06.180067 PMC6396292

[12] Meyer R , Patel AM , Rattana SK , Quock TP , Mody SH . Prescription opioid abuse: a literature review of the clinical and economic burden in the United States. Popul Health Manag. 2014;17(6):372‐387. 25075734 10.1089/pop.2013.0098 PMC4273187

[13] Brennan F , Carr DB , Cousins M . Pain management: a fundamental human right. Anesth Analg. 2007;105(1):205‐221. 17578977 10.1213/01.ane.0000268145.52345.55

[14] Buchman DZ , Ho A , Illes J . You present like a drug addict : patient and clinician perspectives on trust and trustworthiness in chronic pain management. Pain Med. 2016;17:1394‐1406. 26759389 10.1093/pm/pnv083 PMC4975016

[15] Carvalho AS , Pereira SM , Jácomo A , et al. Ethical decision making in pain management: a conceptual framework. J Pain Res. 2018;11:967‐976. 29844699 10.2147/JPR.S162926 PMC5962306

[16] de Sola H , Salazar A , Dueñas M , Failde I . Opioids in the treatment of pain. beliefs, knowledge, and attitudes of the general Spanish population. Identification of subgroups through cluster analysis. J Pain Symptom Manage. 2018;55(4):1095‐1104. 29248566 10.1016/j.jpainsymman.2017.12.474

[17] Ferreira M , Verloo H , Mabire C , Vieira MMS , Marques‐Vidal P . Psychometric evaluation of the French version of the questionnaire attitudes towards morphine use; a cross‐sectional study in Valais, Switzerland. BMC Nurs. 2014;13(1):1‐8. 24406097 10.1186/1472-6955-13-1 PMC4029768

[18] Brooks EA , Unruh A , Lynch ME . Exploring the lived experience of adults using prescription opioids to manage chronic noncancer pain. Pain Res Manag. 2015;20(1):15‐22. 25562838 10.1155/2015/314184 PMC4325885

[19] Von Korff M , Kolodny A , Deyo RA , Chou R . Long‐term opioid therapy reconsidered. Ann Emerg Med. 2011;155(5):325‐328. 10.1059/0003-4819-155-5-201109060-00011 PMC3280085 21893626

[20] Sullivan MD , Von Korff M , Banta‐Green C , Merrill JO , Saunders K . Problems and concerns of patients receiving chronic opioid therapy for chronic non‐cancer pain. Pain. 2010;149(2):345‐353. 20334974 10.1016/j.pain.2010.02.037 PMC3318978

[21] Rosenblum A , Marsch L , Herman J , Russell KP . Opioids and the treatment of chronic pain: controversies, current status, and future directions. Exp Clin Psychopharmacol. 2008;16(5):405‐416. 18837637 10.1037/a0013628 PMC2711509

[22] Nordahl Christensen H , Olsson U , From J , Breivik H . Opioid‐induced constipation, use of laxatives, and health‐related quality of life. Scand J Pain. 2016;11:104‐110. 28850447 10.1016/j.sjpain.2015.12.007

[23] Jiménez JS , Tejedor Varillas A , García CRG , et al. La atención al paciente con dolor crónico no oncológico (DCNO) en atención primaria (AP). 2015 https://www.semfyc.es/wp‐content/uploads/2016/06/DOCUMENTO‐CONSENSO‐DOLOR‐17‐04‐A.pdf. Accessed March 5, 2020.

[24] Cátedra Extraordinaria del Dolor FUNDACIÓN GRÜNENTHAL UNIVERSIDAD SALAMANCA . “Dolor crónico enfermedad en sí misma” XIII Reunión de expertos; 2013.

[25] Dueñas M , Salazar A , Sánchez M , De Sola H , Ojeda B , Failde I . Relationship between using clinical practice guidelines for pain treatment and physicians' training and attitudes toward patients and the effects on patient care. Pain Pract. 2017;18(1):38‐47. 28371291 10.1111/papr.12579

[26] Ljungvall H , Rhodin A , Wagner S , Zetterberg H , Åsenlöf P . “my life is under control with these medications”: an interpretative phenomenological analysis of managing chronic pain with opioids. BMC Musculoskelet Disord. 2020;21(1):1‐14. 10.1186/s12891-020-3055-5 PMC6995209 32005212

[27] St. Marie B . Primary care experiences of people who live with chronic pain and receive opioids to manage pain: a qualitative methodology. J Am Assoc Nurse Pract. 2016;28(8):429‐435. 26799819 10.1002/2327-6924.12342

[28] Frank JW , Levy C , Matlock DD , et al. Patients' perspectives on tapering of chronic opioid therapy: a qualitative study. Pain Med (United States). 2016;17(10):1838‐1847. 10.1093/pm/pnw078 PMC6281041 27207301

[29] Allen MJM , Asbridge MM , MacDougall PC , Furlan AD , Tugalev O . Self‐reported practices in opioid management of chronic noncancer pain: a survey of Canadian family physicians. Pain Res Manag. 2013;18(4):177‐184. 23717824 10.1155/2013/528645 PMC3812188

[30] Antoniou T , Ala‐Leppilampi K , Shearer D , Parsons JA , Tadrous M , Gomes T . “Like being put on an ice floe and shoved away”: a qualitative study of the impacts of opioid‐related policy changes on people who take opioids. Int J Drug Policy. 2019;66:15‐22. 30685650 10.1016/j.drugpo.2019.01.015

[31] Dassieu L , Kaboré JL , Choinière M , Arruda N , Roy É . Painful lives: chronic pain experience among people who use illicit drugs in Montreal (Canada). Soc Sci Med. 2020;246:112734. 31864968 10.1016/j.socscimed.2019.112734

[32] United Nations Office on Drugs and Crime . Global Overview on Drug Demand and Supply: Latest Trends, Cross‐Cutting Issues; 2018 https://www.unodc.org/wdr2018/prelaunch/WDR18_Booklet_2_GLOBAL.pdf

[33] Salazar A , Moreno S , De Sola H , Moral‐Munoz JA , Dueñas M , Failde I . The evolution of opioid‐related mortality and potential years of life lost in Spain from 2008 to 2017. Differences between Spain and the United States. Curr Med Res Opin. 2020;36(2).285–291. 10.1080/03007995.2019.1684251 31635485

[34] Ache dos Santos FA , Barcellos de Souza J, Ledur Antes D , D'Orsi E . Prevalence of chronic pain and its association with the sociodemographic situation and physical activity in leisure of elderly in Florianópolis, Santa Catarina: population‐based study. Rev Bras Epidemiol. 2015;18(1):234‐247. 25651024 10.1590/1980-5497201500010018

[35] Dueñas M , Ojeda B , Salazar A , Mico JA , Failde I . A review of chronic pain impact on patients, their social environment and the health care system. J Pain Res. 2016;9:457‐467. 27418853 10.2147/JPR.S105892 PMC4935027

[36] Timmerman L , Stronks DL , Groeneweg JG , Huygen FJ . Prevalence and determinants of medication non‐adherence in chronic pain patients: a systematic review. Acta Anaesthesiol Scand. 2016;60(4):416‐431. 26860919 10.1111/aas.12697

[37] Graneheim UH , Lundman B . Qualitative content analysis in nursing research: concepts, procedures and measures to achieve trustworthiness. Nurse Educ Today. 2004;24(2):105‐112. 14769454 10.1016/j.nedt.2003.10.001

[38] Slater H , Jordan JE , Chua J , Schütze R , Wark JD , Briggs AM . Young people's experiences of persistent musculoskeletal pain, needs, gaps and perceptions about the role of digital technologies to support their co‐care: a qualitative study. BMJ Open. 2016;6(12):1‐11. 10.1136/bmjopen-2016-014007 PMC5168607 27940635

[39] Ojala T , Häkkinen A , Karppinen J , Sipilä K , Suutama T , Piirainen A . Although unseen, chronic pain is real‐a phenomenological study. Scand J Pain. 2014;6:33‐40. 10.1016/j.sjpain.2014.04.004 29911591

[40] Kempner J . Invisible people with invisible pain: a commentary on “Even my sister says I'm acting like a crazy to get a check”: race, gender, and moral boundary‐work in women's claims of disabling chronic pain. Soc Sci Med. 2017;189:152‐154. 28625413 10.1016/j.socscimed.2017.06.009

[41] Itz C , Huygen F , van Kleef M . A proposal for the organization of the referral of patients with chronic non‐specific low back pain. Curr Med Res Opin. 2016;32(11):1903‐1909. 27499500 10.1080/03007995.2016.1220933

[42] Clarke AE , Shim JK , Mamo L , Fosket JR , Fishman JR . Biomedicalization: technoscientific transformations of health, illness, and U.S. Biomedicine. Am Sociol Rev. 2003;68(4):161‐194.

[43] Meineche‐Schmidt V , Jensen NH , Sjøgren P . Long‐term outcome of multidisciplinary intervention of chronic non‐cancer pain patients in a private setting. Scand J Pain. 2012;3(2):99‐105. 29913771 10.1016/j.sjpain.2011.10.002

[44] World Health Organization (WHO) . Health literacy In: The Solid Facts. Copenhagen; 2013:1‐68.

[45] Linander I , Alm E , Hammarström A , Harryson L . Negotiating the (bio)medical gaze – experiences of trans‐specific healthcare in Sweden. Soc Sci Med. 2017;174:9‐16. 27960120 10.1016/j.socscimed.2016.11.030

[46] Washington TA , Fanciullo GJ , Sorensen JA , Baird JC . Quality of chronic pain websites. Pain Med. 2008;9(8):994‐1000. 18346065 10.1111/j.1526-4637.2008.00419.x

[47] Olaya‐Contreras P , Styf J . Biopsychosocial function analyses changes the assessment of the ability to work in patients on long‐term sick‐leave due to chronic musculoskeletal pain: the role of undiagnosed mental health comorbidity. Scand J Public Health. 2013;41(3):247‐255. 23361388 10.1177/1403494812473380

[48] Monsivais DB . Decreasing the stigma burden of chronic pain. J Am Assoc Nurse Pract. 2013;25(10):551‐556. 24170487 10.1111/1745-7599.12010

[49] Finlay KA , Elander J . Reflecting the transition from pain management services to chronic pain support group attendance: an interpretative phenomenological analysis. Br J Health Psychol. 2016;21(3):660‐676. 27230704 10.1111/bjhp.12194

[50] Jamison RN , Virts KL . The influence of family support on chronic pain. Behav Res Ther. 1990;28(1):283‐287. 2222385 10.1016/0005-7967(90)90079-x

[51] Kowal J , Wilson KG , McWilliams LA , Péloquin K , Duong D . Self‐perceived burden in chronic pain: relevance, prevalence, and predictors. Pain. 2012;153(8):1735‐1741. 22703692 10.1016/j.pain.2012.05.009 PMC3999031

[52] Bäckryd E . “Professional helper” or “helping professional?” the patient–physician relationship in the chronic pain setting, with special reference to the current opioid debate. J Contin Educ Health Prof. 2016;36(2):133‐137. 27116642 10.1097/CEH.0000000000000062

